# Collaborative optimization of depot location, capacity and rolling stock scheduling considering maintenance requirements

**DOI:** 10.1038/s41598-024-57902-5

**Published:** 2024-03-27

**Authors:** Qingwei Zhong, Yingxue Yu, Yiru Huang, Wenxin Li, Yongxiang Zhang, Xu Yan

**Affiliations:** 1 College of Air Traffic Management, Civil Aviation Flight University of China, Guanghan, 618307 China; 2 Hubei Key Laboratory of Power System Design and Test for Electrical Vehicle, Hubei University of Arts and Science, Xiangyang, 441053 China; 3 School of Transportation and Logistics, Southwest Jiaotong University, Chengdu, 611756 Sichuan China; 4 National United Engineering Laboratory of Integrated and Intelligent Transportation, Chengdu, 611756 Sichuan China

**Keywords:** Depot location and capacity, Rolling stock scheduling, Maintenance requirements, Mixed integer programming, Applied mathematics, Civil engineering

## Abstract

Generally, when optimizing a rolling stock schedule, the locations of the depots, or places in the network where the composition changes and maintenance occurs, are assumed known. The locations where maintenance is performed naturally influence the quality of any resulting rolling stock schedules. In this paper, the problem of selecting new depot locations and their corresponding capacities is considered. A two-stage mixed integer programming approach for rolling stock scheduling with maintenance requirements is extended to account for depot selection. First, a conventional flow-based model is solved, ignoring maintenance requirements, to obtain a variety of rolling stock schedules with multiple depot locations and capacity options. Then, a maintenance feasible rolling stock schedule can be obtained by solving a series of assignment problems by using the schedules found in the first stage. The proposed methodology is tested on real-life instances, and the numerical experiments of different operational scenarios are discussed.

## Introduction

In the modern transportation system of China, the railway, especially the high-speed railway (HSR), plays a significant role due to its large capacity, high security and low energy consumption. By the end of 2018, China’s railway mileage reached 131,000  km, of which high-speed railway operating mileage exceeded 29,000 km, accounting for more than 60% of the world’s total high-speed railway mileage^1^. The Chinese high-speed railway (CHSR) network will further expand to 38,000 km by 2025. In such a huge HSR network, approximately 2600 high-speed trains are in operation daily. In order to ensure the safe running of these trains, 10 large depots and more than 50 small depots are scattered throughout the CHSR network as maintenance bases.

In general, the planning of railway operations consists of several interconnected planning problems. Due to the complexity, the problems are often seen as independent stages that can be solved individually. The most important planning problems are line planning, train timetabling, train platforming, rolling stock scheduling, and crew scheduling^2^. Normally, the stage of rolling stock scheduling begins after the train timetable is determined. The timetable outlines services, each described through individual trips. A trip delineates the movement of a train from one station to another at a designated time, characterized by a station of departure, a time of departure, a destination station, and an arrival time. Predictions on the number of passengers for each trip can be made based on past passenger traffic data, enabling the determination of the minimum number of units needed.

Fundamentally, the rolling stock scheduling problem involves assigning so-called compositions to a given timetable. Composition refers to a specific set of rolling stock types coupled together in a specific order. Rolling stock types differ in their physical characteristics (e.g., length, number of seats, etc.) and capabilities (e.g., speed). A railway company usually has multiple rolling stock types and several units of each type. Each rolling stock unit is comprised of several cars. A rolling stock unit is the shortest vehicle that can be assigned; i.e., it cannot be decoupled further. Two units of the same type typically differ in, among other things, their mileage and since maintenance^3^.

In practice, there are several different types of rolling stock units, which lead to many possible compositions. Rolling stock composition changes can happen in some specific places (e.g., depots and some stations). To complete the tasks in one planning horizon, each rolling stock unit will depart from a depot, perform a series of trips, and then return to a depot. Sometimes, deadheading trips and overnight-parking at some stations are needed during this process to ensure the movement continuity. Also, the maintenance requirements (i.e., time and/or distance restrictions) have to be satisfied based on the different states of each rolling stock unit.

Usually, the depot, where the composition change and maintenance occur, is a prerequisite for obtaining a feasible rolling stock schedule that meets the maintenance requirements. The locations and capacities of depots in a railway network naturally influence the maintenance possibilities and ultimately the quality of any resulting rolling stock schedules. Currently, most of the depots in China are located nearby the city center, which makes it difficult to rebuild or expand any existing depots. With the continuous expansion of the CHSR network, the coordination between the locations and capacities of the depots and current transportation needs has declined. Thus, the building of new depots has become the choice of railway planner. As stated by some researchers^2,4^, it does not seem appropriate to consider modifications to the railway infrastructure at the network planning level without at least taking the level of traffic in the near future into consideration.Therefore, it is necessary to study the collaborative optimization of depot location, capacity and rolling stock scheduling with maintenance requirements when rebuilding the depots or selecting new depots.

In this paper, we extend a two-stage mixed integer linear programming (MILP) approach for rolling stock scheduling with maintenance requirements in^3^ to account for depot location and capacity selection. At the first stage, a conventional flow-based model is developed to generate a variety of candidate rolling stock schedules with multiple locations and capacity options (without maintenance requirements). At the second stage, maintenance feasible rolling stock schedules are obtained by solving a series of assignment problems using the schedules found in the first stage. Those two stages can be iteratively performed to find a better rolling stock schedule and the corresponding depot location and capacity options. In summary, this paper discussed the collabrative optimization of depot location, capacity and rolling sotck scheduling considering maintenance reqirements, hoping to provide auxiliary decision-making for the location selection and capacity setting of depots from the perspective of transportation organization.

The rest of this paper is structured as follows. We firstly provide a literature review on the rolling stock scheduling problem in “Literature review”, and “Problem description” sections gives the detailed problem description. In “Mathematical formulation” section, a two-stage MILP model is established and the corresponding solution algorithm is presented. The computation results for a real-world case of China Zhengzhou Railway are shown in “Algorithms” section. Finally, the concluding remarks and future research directions are provided in “Numerical experiments” section.

## Literature review

The rolling stock scheduling problem aims to assign compositions to a set of timetabled trips with a set of operational constraints. Due to its indispensable significance in practice, this problem has attracted the attention of a number of researchers over the past few decades.

The multi-commodity flow problem is a network optimization problem where multiple flows between different source and sink nodes have to be optimized. Also, the multi-commodity flow formulation are quite often used to describe the rolling stock scheduling problem, where a set of different commodities (i.e., rolling stock units with different characteristics) must be routed through a network to satisfy a set of services with different objective values. Ziarati et al.^5^ considered assigning locomotives to train-segments, which was modeled as an arc-flow based multi-commodity flow problem with supplementary constraints. Cordeau et al.^6^ also proposed an arc-based multi-commodity flow model for assigning locomotives and cars to trains in the context of passenger transportation. Brucker et al.^7^ further introduced the depot capacity constraints by formulating an integer arc-based multi-commodity flow problem with a nonlinear objective function. Alfieri et al.^8^ presented an integer arc-based multi-commodity flow model with several additional constraints related to the rolling stock shunting processes (i.e., coupling and decoupling process) at the stations. Fioole et al.^9^ extended an arc-based multi-commodity flow rolling stock model to handle underway combining and splitting of trains. Recently, some researches used path-based multi-commodity flow model to formulate rolling stock scheduling problem, such as Li et al.^10^, Lusby et al.^11^ and Gao et al.^12^. Different from the arc-based multi-commodity flow model, the movements of each individual rolling stock unit were represented by a sequences of interconnected trips in the path-based multi-commodity model. Also, there are some researchers use other methods to describe the rolling stock problem. Abbink et al.^13^ and Cacchiani et al.^14^ solved the problem via the assignment-based problem. Zhao et al.^15^ and Nishi et al.^16^ modelled the rolling stock scheduling problem as a multiple traveling salesman problem.

Recent rapid development of advanced communication and computation technologies enabled a wide range of possibilities for a systematic integration of different railway planning and control aspects across multiple decision layers^17^. The rolling stock scheduling problem is no longer only focused on the rolling stock circulation, but it is also integrated with other issues, such as rolling stock maintenance. Maroti and Kroon^18,19^ developed models to find individual unit routes that adhere to distance and time maintenance requirements based on a fixed rolling stock schedule. Following that, a series of studies focus on rolling stock scheduling with maintenance requirements. Among them, most of the studies only considered single maintenance requirement (i.e., either time or distance), such as Giacco et al.^20^ and Nishi et al.^16^, while some other studies has considered both time and distance maintenance requirements, such as Borndörfer et al.^21^ and Zhong et al.^3^.

To the knowledge of the authors there is only one publication that tries to integrate the depot location into the rolling stock scheduling optimization problem. Canca et al.^4^ proposed a general MILP model in order to design rolling stock circulation plans and determine the number and location of maintenance facilities. However, Canca et al.^4^ focused on Railway Rapid Transit system whose operational rules are quite different from the high-speed railway system considered in our paper, such as the rolling stock (de)coupling process. To the best of our knowledge, the joint depot location, capacity and rolling stock scheduling optimization with maintenance requirements in high-speed railway systems has not been previously studied in the literature. When selecting new depots or rebuilding the existing depots, there is a need for quantitative analyses of the joint depot location, capacity and rolling stock scheduling optimization with maintenance requirements, which can serve as an efficient and reliable evaluation tool for the railway planners.

## Problem description

In general, the train timetable is available before the rolling stock scheduling stage, where the services are defined as a set of timetabled trips with departure stations, departure times, arrival stations and arrival times. In this paper, a minimal number of rolling stock units required for each trip is forecasted according to the historical passenger demand statistics, and each rolling stock unit contains a least 8 cars. According to the operational management requirements of China Railway, all the rolling stock units must be regularly maintained after certain running times or distances. The depot, which is connected to its depot station through a connecting line, is responsible for maintenance and composition changes. Generally, a rolling stock unit departs in the early morning from its depot to the origin (departure) station of its first trip, then it goes back to the depot after taking a sequence of trips at the end of the planning horizon. Throughout the operation process, rolling stocks change their composition according to actual needs. Due to technical reasons in CHSR, only units of the same type can be (de)coupled at a depot or some specific stations with depot tracks. We refer the interested readers to Zhong et al.^3^ for more details of the rolling stock scheduling problem in China.

**Figure 1:**
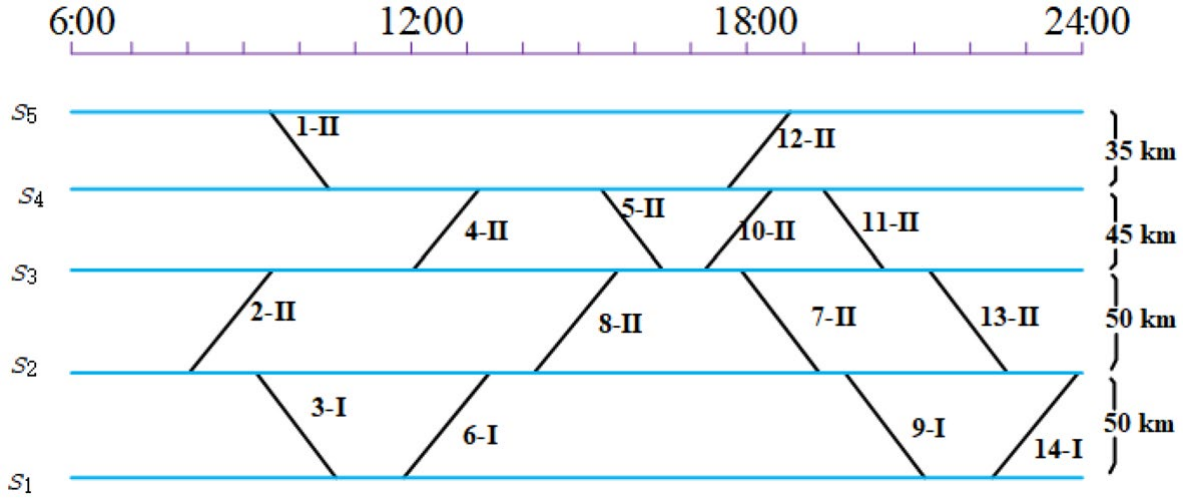
Illustration of the timetable.

Here we use a simple example to illustrate the rolling stock scheduling problem. Assume that there is a CHSR network with 5 main stations and 14 daily trips. The corresponding timetable is shown in Fig. 1, of which the vertical axis represents the stations and the horizontal axis represents the planning time horizon (from 6:00 to 24:00). Each trip has an index (1–14), and the minimum number of rolling stock units required to serve each trips is specified according to the passenger flow forecasted. Further, the predicted passenger flow is converted into the number of cars, where the symbol “I” represents a unit of 8 cars, and “II” represents two units coupled together with 16 cars. We have two types of rolling stock unit. Type AL has only one composition of 16 cars, while Type B has the compositions of 16 or 8 cars due to it can be (de)coupled at the depot and some specific stations. Furthermore, the depot can be built nearby the stations $s_{2}$ and $s_{5}$.

A rolling stock dispatcher is responsible for creating a rolling stock schedule based on the information provided in the timetable shown in Fig. 1. In this process, if two trips are consecutively performed, then they can be formed into a connection. Assuming we have two trips, trip A and trip B, to connect them as a connection, they must meet the following conditions: (1) the terminal station of trip A must be the same as the originating station of trip B; (2) the difference between the arrival time of trip A and the departure time of trip B must be greater than the minimum operating time allowed for the rolling stock at trip B (e.g., loading and unloading passengers, refilling water, etc.). (3) Assess whether there are conditions for changing the composition. At present, there are 2 kinds of cases, the first case: if the terminal station of trip A and the departure station of trip B are the same, but the station does not have the line directly connected to a depot, then it is not possible to carry out composition changes (i.e. the former and the latter must be consistent with the same composition).The second situation: If the terminal station of the current trip A is the same as the departure station of trip B, and the station is on a line directly connected to a depot, then the types of rolling stock of the preceding and subsequent trips must be the same, but the number of rolling stock units can be different. If the number of rolling stock units is different, the dwell time in Condition 2) above must be greater than the minimum permitted operational time for composition changes. Based on the connections, the timetable information in Fig. 1 can be converted into a rolling stock connection graph.

**Figure 2:**
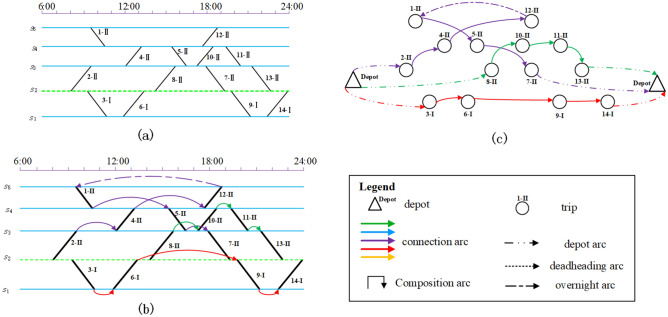
Schedule 1 with one depot near station $s_{2}$.

**Figure 3:**
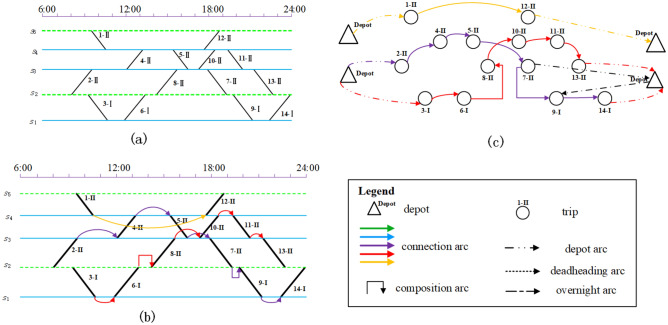
Schedule 2 with two depot near stations $s_{2}$ and $s_{5}$.

If the railway planner decides to build a depot near the station $s_{2}$, which is shown as the green dashed horizontal line in Fig. 2. Trip 1–II does not have a previous trip and not connected with any depot directly. There may be two kinds of ways to serve trip 1–II. One is deadheading units from the depot (near station $s_{2}$) to station $s_{5}$, while the other way is directly using the units which parked nearby the station $s_{5}$ yesterday night. Note that if the station $s_{5}$ is far away from the depot (station $s_{2}$), there might be not enough time to deadhead units before the departure time of trip 1–II. A feasible rolling stock schedule is illustrated in Fig. 2. From the schedule, we can see there are no composition changes in the given trip sequences, thus both units of type B and type AL can be used in this schedule. Trip 1–II is severed by the rolling stock units staying overnight at station $s_{5}$ on the first day, which means it will take 2 days to complete all the trips. According to the operation rules that no shortage of seats is allowed, there are three possible compositions for the third circulation path(a set of trip sequences with a specified order).

If the railway planner decides to build two depots nearby the stations $s_{2}$ and $s_{5}$, i.e., the two green dashed lines in Fig. 3. Different from the schedule in Fig. 2, there are two circulation paths that involve composition changes. Therefore, only units of type B can be used to serve those two circulation paths. With the new depot nearby the station $s_{5}$, trip 1–II can be severed by the units without overnight parking or units with long deadheading distance. The individual trip sequences are also given in (c) of Fig. 3 which naturally converge (diverge) at the coupling (decoupling) location.

**Figure 4:**
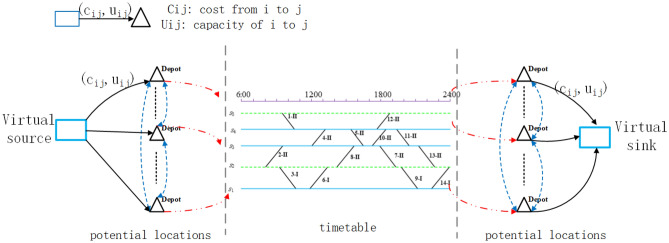
Example of a rolling stock expand connection graph.

**Figure 5:**
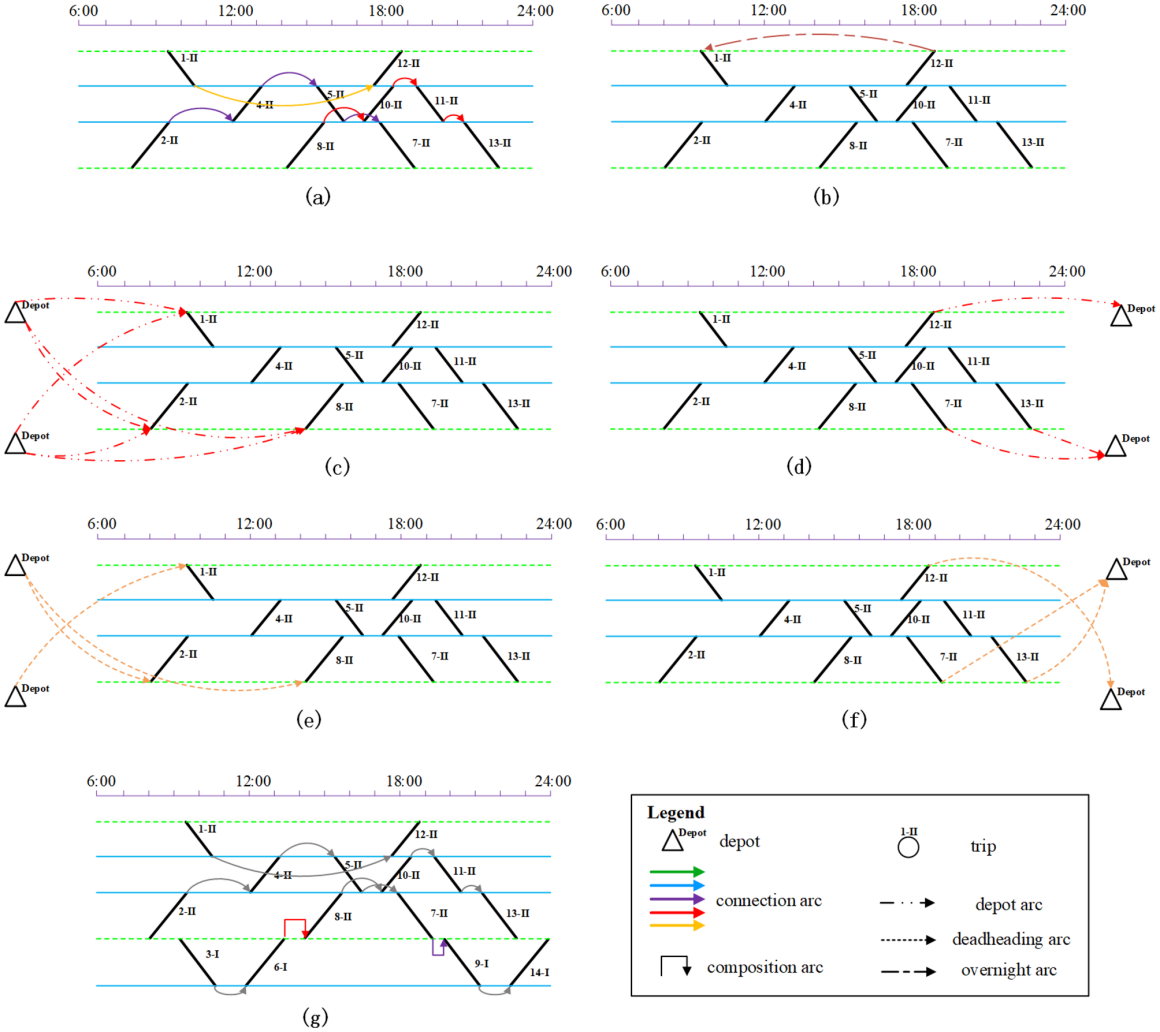
Examples of different arcs.

As shown in Figs. 2 and 3 above, before establishing the mathematical model, it is first necessary to construct a directed graph of rolling stock connection network based on the information of timetable, in which the points represent each trip and the virtural source/sink (as shown in Fig. 4) , and the edges represent the different arcs in the graph (as shown in Fig. 5).

In practice, each unit may have different initial states (i.e., accumulative running times and distance) before the operation period. A circulation path can be assigned to a unit only if its times and mileages consumption is less than the remaining times and mileages of the unit. The level one maintenance requirements of the CHSR is considered in this paper, which specify all units need to go through a maintenance check every 2 days or when their accumulated running mileages exceed 5500 km since the last check. When preparing a rolling stock schedule, both the depot capacity and rolling stock maintenance requirements have to be satisfied. Depots with different locations and capacities will lead to various maintenance feasible rolling stock schedules. Thus, the rolling stock scheduling problem considered in this paper is assigning a maintenance feasible circulation path with a sequences of trips to each individual unit with multiple depot location and capacity options.

## Mathematical formulation

In this section, we present the mathematical models and the corresponding two-stage solution algorithm. Some assumptions are first introduced to facilitate the modeling process.

### Assumptions

1. The timetable is known before scheduling the rolling stock units.
2. Not all stations can be used for overnight parking, to be determined by dispatcher.
3. Deadheading rolling stock units is possible.
4. All rolling stock units are initially located at one dummy depot, and they will be relocated to any existing or built depots during the optimization process.
5. To meet the flexible composition change requirements and maintenance needs in practice, all rolling stock units must go through a coupling and a decoupling procedure at the start and end stations of each circulation path.
6. Depots and their corresponding depot stations can accommodate all kinds of composition changes.

Our approach is based on the idea of decomposing the problem down into two distinct components, which are then solved separately. In the first stage, a multi-commodity flow model considering depot location and capacity is solved first to generate multiple candidate rolling stock schedules. Then, through a path searching algorithm, the candidate schedules are converted to a set of possible circulation paths for each unit. After that, the maintenance requirements for each unit are checked in stage 2 using the sets of the possible circulation paths of each unit as input. The above two-stage model will be described in “Stage 1: multi-commodity flow model considering depot location and capacity” and “Stage 2: trip sequence assignment model” section, respectively. We also designed an iterative optimization algorithm framework to solve the problem, which will be described in “Algorithms” section.

**Table 1:** Definitions of sets, indices, parameters and variables.

	Description
Notation	
*S*	Set of stations
*D*	Set of depots
${{D}_{new}}$	Set of back up depots in the railway network
*P*	Set of rolling stock unit types
*C*	Set of all compositions
*meT*	Set of timetabled trips
*deadheadT*	Set of deadheading trips
*sourceT*	Set of deadheading trips that connect depot and the corresponding depot station,$sourceT \subset deadheadT$
*overT*	Set of all overnight trips
*T*	Set of all trips,$T=deadheadT\cup meT\cup overT$
*Cnn*	Set of all connections
$s_{r}$	The first trip of connection $r\in Cnn$
$t_{r}$	The second trip of connection $r\in Cnn$
$g_{r}$	The station at which connection $r\in Cnn$ takes place
$C_{c,c'}^{r}$	Set of allowable composition changes for connection *r*.This set contains composition *c* and composition $c'$ are allowed for first trip $s_{r}$ and second trip $t_{r}$ respectively
$C_{r,c,c'}^{s}$	The cost incurred when changing from composition *c* to $c'$ on connection *r*
$C_{t,c}^{o}$	The operational cost of using composition $c\in C$ on trip $t \in T$
$couple_{c,c'}^{p}$	The number of units of type $p\in P$ that are coupled when changing from composition *c* to $c'$
$decouple_{c,c'}^{p}$	The number of units of type $p\in P$ that are decoupled when changing from composition *c* to $c'$
$tDecoupling_{r}$	Decoupling time necessary on connection *r*. This is the amount of time that must elapse before a decoupled unit can be used again
$tCoupling_{r}$	Coupling time necessary on connection *r*. The amount of time necessary to perform a coupling
*Time*	The planning horizon in minutes
$Nroll_{p}$	The total number of type $p\in P$ rolling stock units
*Ndepot*	The maximum number of depots in the railway network
${{O}_{c}}$	The combined operating cost
$Maxtrack_{d}$	The maximize number of tracks that a depot can have based on the accrual operation conditions
$C_{d}^{trc}$	The cost of building one track at depot $d \in D_{new}$
$C_{d}^{dop}$	The cost of opening one depot at the railway network $d\in D_{new}$
Variable	
$y_{t}^{c}$	Binary decision variable indicating whether or not composition $c\in C$ is used on trip $t \in T$
$i_{d}^{p}$	Non-negative integer decision variable stating the imbalance of unit type $p\in P$ at depot $d\in D$
$X_{c,c'}^{r}$	Binary decision variable deciding whether composition *c* and $c'$ are used for trip $s_{r}$ and $t_{r}$ respectively
$v1_{r}^{p},v2_{r}^{p}$	Non-negative integer decision variables which represent the number of unit type $p\in P$ coupled and decoupled in the connections $r\in Cnn$
$h_{r,p}^{d},g_{r,p}^{d}$	Binary decision variables deciding whether the coupled and decoupled unit type $p \in P$ in the connection $r\in Cnn$ is from depot $d \in D$ respectively
$storage_{p,d}^{time}$	Non-negative decision variable stating the inventory of type $p \in P$ at depot $d \in D$ at any time $time\in Time$
$Mil{{e}^{de}}$	The total deadhead mileages of the schedule
$Tim{{e}^{o}}$	The total overnighting times of the schedule
$starstor_{d}^{p}$	Non-negative integer decision variable which represents the number of unit type $p \in P$ that are parked in depot $d \in D$ at the beginning of the planning horizon
$op_{d}^{trc}$	Non-negative integer decision variable which represents the number of tracks in depot *d*
$o{{p}_{d}}$	Binary decision variable indicating the depot $d\in D_{new}$ is open or not

### Stage 1: multi-commodity flow model considering depot location and capacity

Table 1 gives the definitions of sets, indices, parameters and variables that are needed to formulate the mathematical models.

Note that the railway planner is expected to generate a maintenance feasible schedule with the minimum cost and the corresponding depot locations and capacities are selected under the capital budget constraints. To produce a unified evaluation function, we particularly introduce a linear function $G(x)=Gx$ to convert the construction cost into the generalized operating cost^22^, where *G* is a constant. Therefore, the objective function in Eq. (1) consists of two parts, including the combined operating cost and construction cost.

1 $$\begin{aligned} \min \ {{Z}_{1}}={{O}_{c}}+G\cdot \sum \limits _{d\in {{D}_{new}}}\left( C_{d}^{trc}\cdot op_{d}^{trc}+C_{d}^{dop}\cdot o{{p}_{d}}\right) \end{aligned}$$

The trade-off relationship between the construction cost and the combined operating cost can be explored by setting the weight *G* to different values. Equation (2) specifies the combined operation cost *Oc* in the objective function.

2 $$\begin{aligned} \begin{aligned} {{O}_{c}}=\sum \limits _{t\in T}{\sum \limits _{c\in C}{C_{t,c}^{o}\cdot y_{c}^{t}}}+\sum \limits _{r\in Cnn}{\sum \limits _{c\in C}{\sum \limits _{c'\in C}{C_{r,c,c'}^{s}\cdot X_{c,c'}^{r}}}} +\sum \limits _{p\in P}{\sum \limits _{d\in D}{\eta \cdot i_{d}^{p}}}+\alpha \cdot Tim{{e}^{o}}+\beta \cdot Mil{{e}^{de}} \\ \end{aligned} \end{aligned}$$

In Eq. (2), $\eta$ represents The penalty for storage imbalance at the end of the planning horizon, $\alpha$ and $\beta$ represent the penalty parameters for overnight time and deadhead mileage. The variable $Tim{{e}^{o}}$ represents the total overnight times of the schedule, which is computed in Eq. (3).

3 $$\begin{aligned} Tim{{e}^{o}}=\sum \limits _{ {\begin{matrix} r\in Cnn \\ {{s}_{r}}\in overT \end{matrix}}}{\sum \limits _{c\in C}{\sum \limits _{c'\in C}{X_{c,c'}^{r}\cdot overtim{{e}_{{{s}_{r}}}}}}} \end{aligned}$$

where the $overtime_{t}$ means the overnight time of trip *t*. Similarly, Eq. (4) gives the running distance of all deadhead trips used in the solution.

4 $$\begin{aligned} \begin{aligned} Mil{{e}^{de}}&= \sum \limits _{{\begin{matrix} r\in Cnn: \\ {{s}_{r}}\in deadheadT \end{matrix}}}{\sum \limits _{c\in C}{\sum \limits _{c'\in C}{X_{c,c'}^{r}\cdot lengt{{h}_{{{s}_{r}}}}\cdot {{N}_{c}}+}}}\sum \limits _{{\begin{matrix} r\in Cnn: \\ {{t}_{r}}\in deadheadT, \\ {{B}_{{{t}_{r}}}}\in D \end{matrix}}}{\sum \limits _{c\in C}{\sum \limits _{c'\in C}{X_{c,c'}^{r}\cdot lengt{{h}_{{{t}_{r}}}}}}}\cdot {{N}_{c'}} \\&\quad +\sum \limits _{{\begin{matrix} r\in Cnn: \\ {{s}_{r}},{{t}_{r}}\notin sourceT \end{matrix}}}{\sum \limits _{d\in D}{\sum \limits _{p\in P}{\sum \limits _{{\begin{matrix} t\in T: \\ {{A}_{t}}=d, \\ {{B}_{t}}={{B}_{{{s}_{r}}}} \end{matrix}}}{g_{r,p}^{d}}}}}\cdot lengt{{h}_{t}}+\sum \limits _{{\begin{matrix} r\in Cnn: \\ {{s}_{r}},{{t}_{r}}\notin sourceT \end{matrix}}}{\sum \limits _{d\in D}{\sum \limits _{p\in P}{\sum \limits _{{\begin{matrix} t\in T: \\ {{B}_{t}}=d, \\ {{A}_{t}}={{B}_{{{t}_{r}}}} \end{matrix}}}{h_{r,p}^{d}}}}}\cdot lengt{{h}_{t}} \\ \end{aligned} \end{aligned}$$

where the $length_{t}$ means the length of trip *t*, $N_{c}$ represents the number of rolling stock units in composition *c*, $A_{t}$ and $B_{t}$ refer to the arrival and departure stations (depots) of trip *t* respectively.

To obtain a feasible rolling stock schedule, the flow-based model is subject to a number of constraints. First of all, each timetabled trip must be covered by exactly one composition in the planning horizon. Due to the reason that some overnight parking activities are usually unavoidable, each trip is replicated twice and it will appear both on the first and second days.Specifically, the set of trips that need to be completed on the first day is defined as $t \in {T^1}$, the corresponding set of trips for the next day is $t \in {T^2}$ (${T^1}$ and ${T^2}$ are exactly the same except for the time). It should be noted that in the constructed rolling stock connection network, by controlling the added depot arcs(entry and exit), it can be ensured that all rolling stock depart from the first day. Therefore, in order to complete all the trips that need to be performed in the timetable within a day, the corresponding trains in ${T^1} \cup {T^2}$ must be performed by one and only one rolling stock. Equation (5) enforces that exactly one of the two trips must receive a valid composition.

5 $$\begin{aligned} \sum \limits _{c \in C} {y_t^c} + \sum \limits _{c \in C} {y_{t'}^c} = 1\begin{array}{*{20}{c}} {}&{\forall t \in {T^1},t'} \end{array} \in {T^2} \end{aligned}$$

Equation (6) specifies that both deadheading trips and overnighting trips are not necessarily covered by a composition.

6 $$\begin{aligned} \sum \limits _{c\in C}{y_{t}^{c}}\le 1\begin{matrix} {} &{} \forall t\in deadheadT\bigcup overT \\ \end{matrix}:{{A}_{t}},{{B}_{t}}\notin D \end{aligned}$$

To ensure that feasible and consistent rolling stock compositions are assigned to the trips, Eqs. (7) and (8) are required to monitor the composition transitions between consecutive trips where each trip must receive one of its allowed compositions.

7 $$\begin{aligned} \sum \limits _{{\begin{matrix} r\in Cnn: \\ {{s}_{r}}=t \end{matrix}}}{\sum \limits _{c':(c,c')\in C_{c,c'}^{r}}{X_{c,c'}^{r}}}=y_{t}^{c}\begin{matrix} {} &{} \forall t\in T:{{A}_{t}},{{B}_{t}}\notin D,c\in C \\ \end{matrix} \end{aligned}$$

8 $$\begin{aligned} \sum \limits _{{\begin{matrix} r\in Cnn: \\ {{t}_{r}}=t \end{matrix}}}{\sum \limits _{c':(c',c)\in C_{c',c}^{r}}{X_{c',c}^{r}}}=y_{t}^{c}\begin{matrix} {} &{} \forall t\in T:{{A}_{t}},{{B}_{t}}\notin D,c\in C \\ \end{matrix} \end{aligned}$$

Equation (9) describes that the number of rolling stock units of each type that are initially parked at all depots should be less than the total number of units of this type that are available.

9 $$\begin{aligned} \sum \limits _{d\in D}{starstor_{d}^{p}}\le Nrol{{l}_{p}}\begin{matrix} {} &{} \forall p\in P \\ \end{matrix} \end{aligned}$$

During the operating period, the inventories of different types of rolling stock units at every depot are related to the (de)coupling process, and Eqs. (10) and (11) are introduced to monitor the changes in the number of rolling stock units.

10 $$\begin{aligned} v1_{r}^{p}=\sum \limits _{c\in C}{\sum \limits _{c':(c,c')\in C_{c,c'}^{r}}{X_{c,c'}^{r}}}\begin{matrix} \cdot couple_{c,c'}^{p} &{} \forall r\in Cnn,p\in P \\ \end{matrix} \end{aligned}$$

11 $$\begin{aligned} v2_{r}^{p}=\sum \limits _{c\in C}{\sum \limits _{c':(c,c')\in C_{c,c'}^{r}}{X_{c,c'}^{r}}}\begin{matrix} \cdot decouple_{c,c'}^{p} &{} \forall r\in Cnn,p\in P \\ \end{matrix} \end{aligned}$$

Equation (12) records the number of rolling stock of a certain type at any time in a depot.

12 $$\begin{aligned} \begin{aligned} storage_{p,d}^{time}&=starstor_{d}^{p}-\sum \limits _{{\begin{matrix} r\in Cnn: \\ {{B}_{{{s}_{r}}}}=d, \\ tCouplin{{g}_{r}}\le time \end{matrix}}}{v1_{r}^{p}}+\sum \limits _{{\begin{matrix} r\in Cnn: \\ {{A}_{{{t}_{r}}}}=d, \\ tDecouplin{{g}_{r}}\le time \end{matrix}}}{v2_{r}^{p}} \\&\quad +\sum \limits _{{\begin{matrix} r\in Cnn: \\ {{s}_{r}},{{t}_{r}}\notin sourceT, \\ tDecouplin{{g}_{r}}\le time \end{matrix}}}{g_{r,p}^{d}}-\sum \limits _{{\begin{matrix} r\in Cnn: \\ {{s}_{r}},{{t}_{r}}\notin sourceT, \\ tCouplin{{g}_{r}}\le time \end{matrix}}}{h_{r,p}^{d}} \\&\quad \begin{matrix} {} &{} \forall time\in Time,p\in P,d\in D \\ \end{matrix} \\ \end{aligned} \end{aligned}$$

In Eq. (12), the used rolling stock units are divided into two parts, i.e., units that are (de)coupled at the depot and those that are (de)coupled at stations. Equations 13 and 14 are proposed for calculating the number of rolling stock units (de)coupled at stations. Note that those tow equations can help us to identify the corresponding depot of (de)coupled units.

13 $$\begin{aligned} \sum \limits _{d\in D}{g_{r,p}^{d}}=v2_{r}^{p}\begin{matrix} {} &{} \forall r\in Cnn:{{s}_{r}},{{t}_{r}}\notin sourceT;p\in P \\ \end{matrix} \end{aligned}$$

14 $$\begin{aligned} \sum \limits _{d\in D}{h_{r,p}^{d}}=v1_{r}^{p}\begin{matrix} {} &{} \forall r\in Cnn:{{s}_{r}},{{t}_{r}}\notin sourceT;p\in P \\ \end{matrix} \end{aligned}$$

The imbalance of units can be expressed by the difference in the number of rolling stock units in a depot at the beginning and end of the planning horizon. In addition to the constraints above, there are also constraints related to the depot location and capacity.

For all the depots, Eq. (15) enforces that the depot tracks can be built only when the corresponding depot is opened. In addition, Eq. (16) specifies the maximum number of depots that can be opened in the railway network.

15 $$\begin{aligned} op_{d}^{trc}\le M\cdot \begin{matrix} o{{p}_{d}} &{} \forall d\in D \\ \end{matrix} \end{aligned}$$

16 $$\begin{aligned} \sum \limits _{d\in D}{o{{p}_{d}}}\le Ndepot \end{aligned}$$

For every depot opened in the railway network, Eq. (17) requires that the number of tracks inside the depot should be at least larger than the number of rolling stock units that need to park at this depot before operating.

17 $$\begin{aligned} \sum \limits _{p\in P}{starstor_{d}^{p}}\le op_{d}^{trc}\begin{matrix} {} &{} \forall d\in D \\ \end{matrix} \end{aligned}$$

Due to the limitation of construction budget, Eq. (18) restrict the maximum number of tracks that can be built within each new depot.

18 $$\begin{aligned} op_{d}^{trc}\le {{\begin{matrix} Maxtrac{{k}_{d}} &{} \forall d\in D \\ \end{matrix}}_{new}} \end{aligned}$$

Equation (19) enforces that the number of tracks of one depot should also be larger than the inventory at any time of the planning horizon, which is to make sure there is always enough track for parking the rolling stock units.

19 $$\begin{aligned} \sum \limits _{p\in P}{storage_{p,d}^{time}}\le op_{d}^{trc}\begin{matrix} {} &{} {} \\ \end{matrix}\forall d\in D,time\in Time \end{aligned}$$

Objective function (1) and Eqs. (2)–(19) form the model of stage 1, which can provide multiple candidate rolling stock schedule with multiple depot location and capacity options.

### Stage 2: trip sequence assignment model

To determine whether a rolling stock schedule generated in the stage 1 is feasible with respect to maintenance requirements, an MILP that assigns individual circulation path that consists of a set of trip sequences to the units can be defined. This model is termed as Trip Sequence Assignment Model (TSAM).

One Binary decision variable $j_{roll}^{l}$ is introduced here, indicating whether rolling stock $roll\in Rolling$ is used for the individual trip sequences $l\in {{L}_{roll}}$. The other notations used in the TSAM are listed as follows: *Rolling* represents the set of all rolling stock units, $roll\in Rolling$; *L* is the set of all possible individual trip sequences; ${{L}_{roll}}$ indicates the set of possible individual trip sequences for a single rolling stock unit $roll\in Rolling$ considering maintenance requirements; $assCos{{t}_{roll}}$ expresses the artificial cost of a single rolling stock unit $roll\in Rolling$; $a_{l}^{t}$ is a binary parameter indicating whether or not trip $t\in T$ is contained in trip sequence $l\in {{L}_{roll}}$; Integer parameter ${{b}_{t}}$ representing the number of units needed by trip $t\in T$. Note that this parameter is decided by the values of binary variable $y_{t}^{c}$ in the arc-flow model of stage 1.

If multiple feasible solutions exist for the TSAM, we are interested in the one that use the least number of rolling stock units. Thus, we introduce an artificial activation cost for each rolling stock unit. Activating a recently maintained unit is seen to be an expensive option and therefore incurs a higher cost. The objective function of stage 2 is defined in Eq. (20).

20 $$\begin{aligned} \min \ z=\sum \limits _{l\in L}{\sum \limits _{roll\in Rolling}{j_{roll}^{l}\cdot assCos{{t}_{roll}}}} \end{aligned}$$

There are two main types of constraints in the MILP model. Equation (21) ensures that the number of rolling stock units assigned to each trip is consistent with the solution to the arc-flow model. Constraint (22) specifies that each unit can receive at most one trip sequence.

21 $$\begin{aligned} \sum \limits _{roll\in Rolling}{\sum \limits _{l\in {{L}_{roll}}}{a_{l}^{t}}}\cdot j_{roll}^{l}={{b}_{t}}\begin{matrix} {} &{} \forall t\in meT \\ \end{matrix} \end{aligned}$$

22 $$\begin{aligned} \sum \limits _{l\in {{L}_{roll}}}{j_{roll}^{l}\le 1}\begin{matrix} {} &{} \forall roll\in Rolling \\ \end{matrix} \end{aligned}$$

For a given unit $roll\in Rolling$, the set ${{L}_{roll}}$ can be generated according to the rolling stock schedule obtained in the stage 1. During the planning horizon, a trip sequence *l* in ${{L}_{roll}}$ assigned to each unit should satisfy its time and distance restrictions (level one maintenance requirements mentioned before) in Eqs. (23) and (24) respectively.

23 $$\begin{aligned} \begin{matrix} \sum \limits _{t}{a_{l}^{t}\cdot lengt{{h}_{t}}+Slengt{{h}_{roll}}\le 5500} &{} \begin{aligned} &{} \forall roll\in Rolling,l\in {{L}_{roll}}: \\ &{} \begin{matrix} {} &{} {} \\ \end{matrix}Typ{{e}_{roll}}={{l}_{type}}\bigcap {{l}_{depot}}=Depo{{t}_{roll}} \\ \end{aligned} \\ \end{matrix} \end{aligned}$$

24 $$\begin{aligned} \begin{matrix} {{l}_{time}}+Stim{{e}_{roll}}\le 2880 &{} \begin{aligned} &{} \begin{matrix} {} &{} \begin{matrix} {} &{} \begin{matrix} {} &{} {} \\ \end{matrix} \\ \end{matrix} \\ \end{matrix}\forall roll\in Rolling,l\in {{L}_{roll}}: \\ &{} \begin{matrix} {} &{} \begin{matrix} {} &{} \begin{matrix} {} &{} \begin{matrix} {} &{} {} \\ \end{matrix} \\ \end{matrix} \\ \end{matrix} \\ \end{matrix}Typ{{e}_{roll}}={{l}_{type}}\bigcap {{l}_{depot}}=Depo{{t}_{roll}} \\ \end{aligned} \\ \end{matrix} \end{aligned}$$

In Eqs. (23) and (24), $Stime_{roll}$ and $Slength_{roll}$ denote the initial accumulated running times and distance of rolling stock units $roll\in Rolling$; $Typ{{e}_{roll}}$ and $Depo{{t}_{roll}}$ indicate the type of rolling stock units $roll\in Rolling$ and the initial parking depot of $roll\in Rolling$. $l_{type}$, $l_{depot}$, $l_{time}$ represent the required type of rolling stock unit, required departure depot and the running time of trip sequence *l*, respectively.

If a feasible solution to the TSAM is found, a maintenance feasible rolling stock schedule can be obtained. It could be necessary to consider several TSAM problems as there is no guarantee that the first one is optimal. In what follows, we provide an overview of the full two-sage algorithm.

## Algorithms

**Figure 6:**
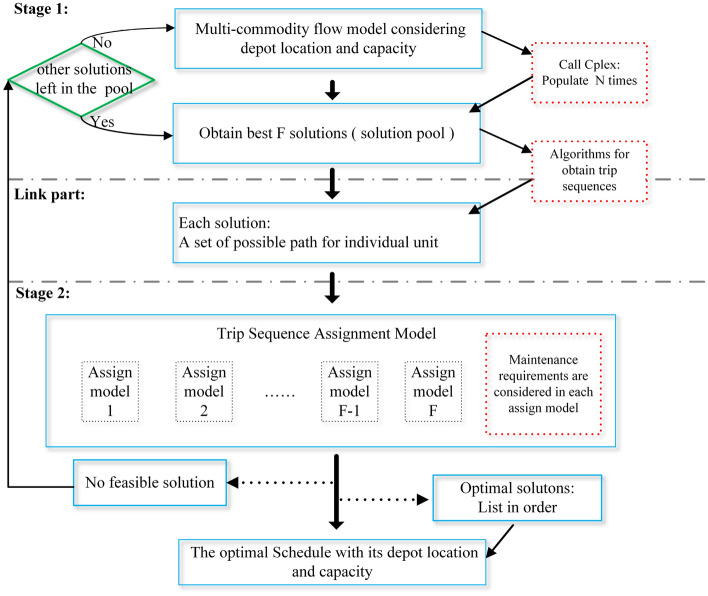
Flow diagram for the proposed method.

In the complex high-speed rail network with multiple depots and multiple types of rolling stock, the study of collaborative optimization of depot location, capacity and rolling stock scheduling, taking into account maintenance requirements, is a difficult task. The problem is difficult to find an optimal or near-optimal solution in a short time, as many practical constraints have to be considered, such as operational safety and daily maintenance constraints of rolling stock. As mentioned above, it can be solved using an iterative optimization algorithm combined with a two-stage model: in the first stage, a multi-commodity flow model (based on Fioole et al.^9^ and Haahr et al.^23^) , taking into account depot location and capacity, is first solved to generate several candidate rolling stock schedules; then, using a path search algorithm, the candidate schedules are converted into a set of possible circulation paths for each unit; then, in the second stage, the maintenance requirements for each unit are checked using the sets of possible circulation paths for each unit as input. Finally, the solution that corresponds to the optimal value of the objective function is selected from those solutions that satisfy the daily maintenance constraints. Although this iterative solution algorithm, which splits the whole problem into two stages does not guarantee a globally optimal solution to the entire problem, it does provide a fast way to find a feasible set^3^. The solution framework of the proposed two-stage algorithm is shown in Fig. 6.

In Fig. 6, through the multi-commodity flow model considering depot location and capacity, obtain N rolling stock schedules. This is done by calling the populate function of the commercial optimization solver CPLEX. The N feasible solutions are stored in the solution pool, then the N solutions are sorted according to the value of the objective function and the top F solutions are selected to proceed to the next step. For the selected F solutions, a designed searching algorithm transforms rolling stock schedules into a set of possible trip sequences of a rolling stock unit, and uses this set of possible trip sequences as input to the second stage. This is shown as the link part is the connection between stages 1 and 2, which is very important in the whole framework. In Fig. 6, the detailed algorithmic steps for generating all of the possible trip sequences according to the rolling stock schedules obtained in the stage 1 is illustrated in detail. The second stage of the model, i.e., the trip sequences assignment model with maintenance constraints, is then used to assign specific rolling stock units to paths in the set of feasible paths, taking into account the initial state of each alternative rolling stock unit and the constraints on the daily maintenance requirements. At this point, if the second stage model has a feasible solution, it has been shown to have found a rolling stock schedule that satisfies the daily maintenance constraints with a depot location under a capacity constraint. If more than one feasible solution exists, these solutions are ranked in terms of the first-stage. The objective function values are sorted from smallest to largest, and then the solution with the smallest objective function value is selected for output. When there are no feasible solutions for the model in the second stage, it is necessary to return to the solution pool and select another F solutions from it in turn to enter the link part. If no more solutions exist in the solution pool, it is necessary to return to the first stage model to force it to generate more solutions and put them into the solution pool, and then repeat all the steps in turn.

The whole framework is programmed in C#, in which the commercial solver CPLEX 12.8.0 is called to solve the MILP problems.

**Algorithm 1:**
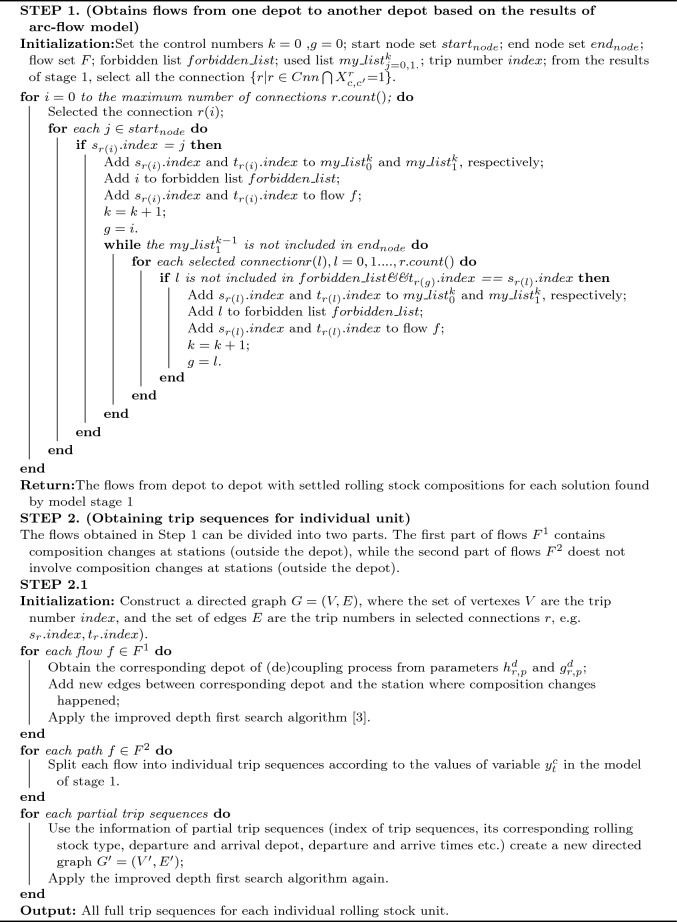
Full trip sequences searching algorithms

## Numerical experiments

### Network description and experimental setup for the real-life instances

**Figure 7:**
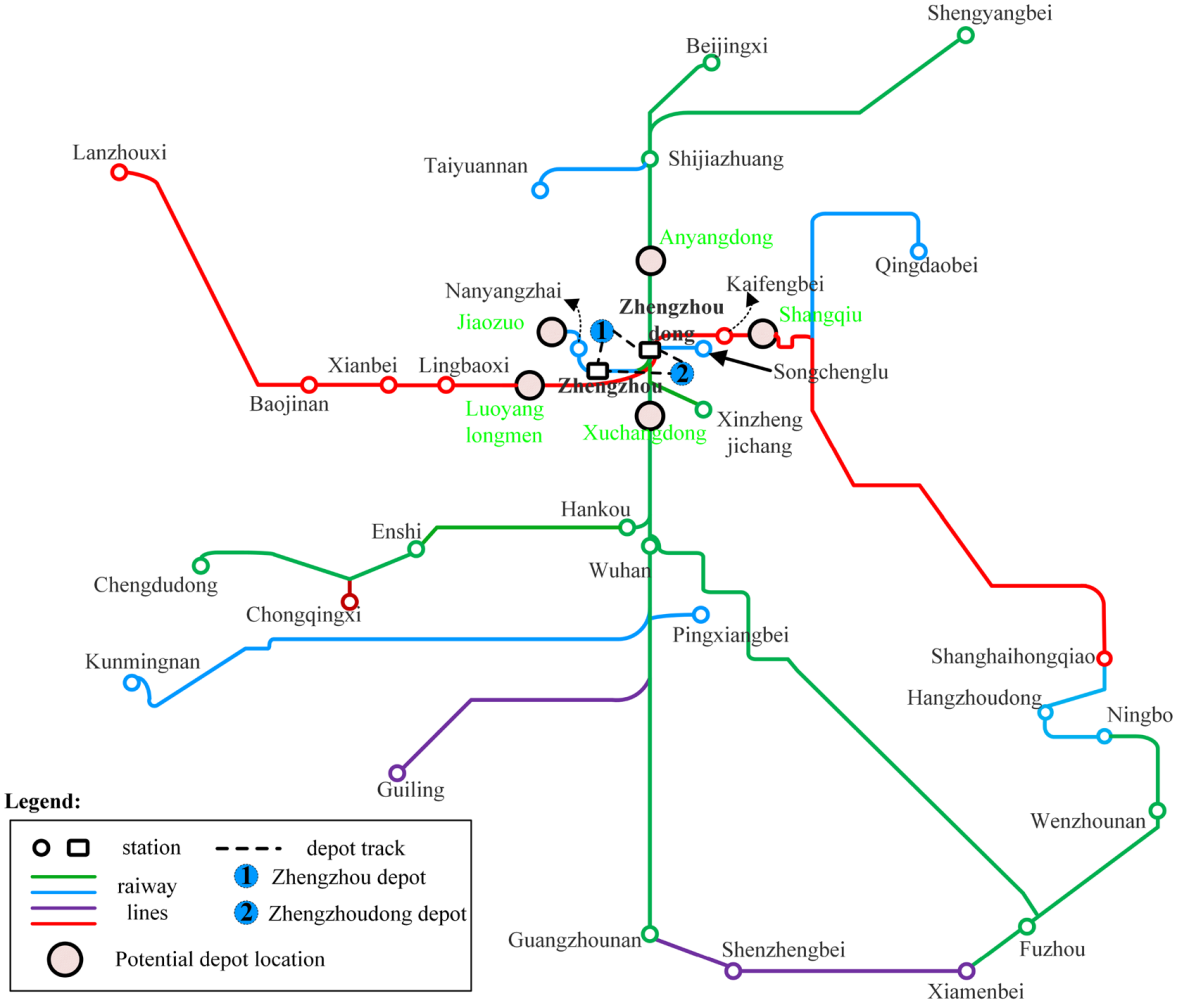
Sketch of China Zhengzhou Railway network.

A real-life instance provided by the Zhengzhou Railway of CHSR is used to illustrate the proposed methodology. An overview of this network is provided in Fig. 7. There are 36 main stations, 2 original depots (i.e., Zhengzhou depot and Zhengzhoudong depot in blue dots) and 5 potential depot locations. Four different types of rolling stock units are operated in this network, which consists of 7 compositions (for more detail of each composition, we refer the readers to^3^. Each rolling stock unit is assumed to have a known accumulated mileages and running times since its last maintenance check. Two timetable data-sets are used, where timetable 1 consists of 377 trips (2017–2018) and timetable 2 contains 490 trips (2018–2019) in a planning horizon of 2 days.

The penalty parameters $\alpha$ and $\beta$ for deadhead mileage and overnight time are set to 1 and 4, respectively. According to existing research, the storage imbalance penalty is set to 100,000 to ensure the balance of the rolling stock inventory in each depot^3,23^. The construction cost is converted into daily expense, where the cost of 1000 RMB is needed for either selecting a new depot or building one depot track. In order to reduce the impact of construction costs on the rolling stock schedule, the parameter *G* inEq. (1) is set to 0.005.Other parameters related to the operation costs are confirmed through the discussions with the railway dispatchers at the Zhengzhou Railway.

We use C# to program the proposed two-stage algorithm, in which the commercial solver CPLEX 12.8.0 is called to solve the MILP models. The solution pool intensity parameter in CPLEX is set to 3, the maximum number N of MILP solutions generated for the solution pool during each call to the populate procedure is set to 500, and the number of best schedules F chosen from the first stage equals to 50. A desktop computer with i7-7700 @ 3.6 GHz CPU and 32.0 GB RAM is used for running the tests.

### Computational results

Two kinds of operating scenarios are considered in our computation tests. For each scenario, the cases with the maximum number of depots ranging from 2 to 7 are tested.

#### Scenario 1

For scenario 1, the two original depots are opened without limiting their capacities. Meanwhile, new depots can be opened at the potential locations with a maximize budget (5 depot track budget for each potential depot). The key statistics of different cases are given in Table 2 and the corresponding depot information is shown in Table 3.

In Table 2, the first column “Data” indicates data-set number of these cases, the second column “Index” is the index of each case. Columns “Time” and “${\mathrm{Depot}}_{\mathrm{num}}$” represent the computation time in seconds and the maximum number of depots that can be opened. Columns “RS”, “${\mathrm{L}}_{{\rm avg}}$”, “${\mathrm{L}}_{\min}$”, “${\mathrm{L}}_{\max}$”, “${\mathrm{L}}_{{\rm tdh}}$” and “${\mathrm{L}}_{{\rm adh}}$” refer to the number of rolling stock units used, the average running distance of each unit (km), the minimal running distance of a unit (km), the maximal running distance of a unit (km), the total deadheading distance (km), the average deadheading distance (km) respectively. Columns “OP” and “Obj” indicate the number of units overnighting at stations and total operation costs respectively. In addition to the information mentioned above, the column “${\mathrm{Depot}}_{{\rm use}}$” is the number of depot used in the railway network and other columns in Table 3 are the name abbreviations, e.g., “ZZD” represents the abbreviation of “zhengzhoudong depot”. In Table 3, the symbol “-” means the corresponding depot is not opened, while “0” means the corresponding depot is opened and no depot-track in the depot is occupied during the planning horizon. The “0” in Table 3 indicates that the depot is open and has a connecting line to the main line. During operation, the connecting lines of the depot are available for use, allowing more flexible deadheading of rolling stock. However, there is no need to park any units inside the depot, so the internal tracks are not used, so there is no need to build them (the number of tracks is 0). Case 1 and Case 8 are the manual schedules prepared by the rolling stock dispatcher, i.e. the rows in italics in Tables 2 and 3. The dispatcher prepares the manual schedules by activating the two original depots ZZ and ZZD.

For both data-sets in Table 2, it can be shown that the objective values are decreasing when the maximum number of allowed depots in the railway network is increasing. However, as the number of available depots increases, the decline rate of the objective values becomes smaller, e.g., the objective value of case 7 is even same as that of case 6. Compared with the cases using two original depots, there are about 2.4% of operation cost savings for 1 day timetable. The deadheading mileage has a similar trend to the objective value. It is worth mentioning that, there is a tradeoff relationship between the number of rolling stock units used and the number of overnight parking units. As we can see from cases 3 and 4, the number of rolling stock units used increases when the number of overnight parking units decreases. This is because the overnighting trips can now be served on the day of operation using other rolling stock units from one of the closer depot stations. Table 3 indicates the “LYLM” depot is the first one considered to open, but no depot tracks are needed inside the depot. This means only the deadheading possibilities by opening this depot is enough. For all the cases, it can be seen that the depot “SQ” is important compared to other depots, where 3 depot tracks are needed in the depot “SQ”. The track occupation information of the two original depots has not changed much, which shows the main position of those two original depots.

Overall, computer-aided planning is superior to manual planning. For the situations that are similar to the scenario 1, the railway planner can consider opening the “LYLM” depot to add more deadheading possibilities. If there is more capital budget for construction, the “SQ” depot can also be opened with the depot tracks inside.

**Table 2:** Key Statics for different cases of scenario 1.

Data	Index	Time	${Depot_{num}}$	RS	${L_{avg}}$	${L_{min}}$	${L_{max}}$	${L_{tdh}}$	${L_{adh}}$	OP	Obj
1	*1*	*–*	*2*	*57*	*2435*	*183*	*4898*	*5291*	*92.8*	*13*	*124,471*
	2	176s	2	51	2676.9	401	5363	3191	62.6	13	109,671
	3	917s	3	51	2672.7	453	5419	2975	58.3	13	107,860
	4	852s	4	53	2572.3	465	5134	2993	56.4	11	106,058
	5	740s	5	53	2571.7	364	5314	2971	56.1	11	106,041
	6	594s	6	53	2571.7	421	5134	2961	55.9	11	106,036
	7	580s	7	53	2571.7	421	5134	2961	55.9	11	106,036
2	*8*	*–*	*2*	*70*	*2575.7*	*571*	*5229*	*5240*	*74.8*	*18*	*160,753*
	9	382s	2	66	2714.2	669	5488	4412	66.8	18	145,012
	10	1832s	3	66	2710.4	498	5488	4172	63.2	18	143,177
	11	1831s	4	68	2631.0	654	5488	4194	61.7	16	141,879
	13	1840s	5	68	2630.3	654	5488	4140	60.9	16	141,830
	13	1757s	6	68	2629.9	774	5488	4110	60.4	16	141,805
	14	1831s	7	68	2629.7	654	5488	4110	60.4	16	141,805

Manual schedule values are in [italics].

**Table 3:** Depot and its track occupy information of scenario 1.

Index	${Depot_{use}}$	ZZ	ZZD	SQ	LYLM	XCD	JZ	AYD
*1*	*2*	*10*	*47*	*–*	*–*	*–*	*–*	*–*
2	2	18	33	–	–	–	–	–
3	3	18	33	–	0	–	–	–
4	4	18	32	3	0	–	–	–
5	5	18	32	3	0	0	–	–
6	6	17	33	3	0	0	–	0
7	6	17	33	3	0	0	–	0
*8*	*2*	*7*	*63*	*–*	*–*	*–*	*–*	*–*
9	2	17	49	–	–	–	–	–
10	3	17	49	–	0	–	–	–
11	4	17	48	3	0	–	–	–
12	5	17	48	3	0	0	–	–
13	6	15	50	3	0	0	–	0
14	6	15	50	3	0	0	–	0

Manual schedule values are in [italics].

#### Scenario 2

For scenario 2, we limit the number of depot tracks of the two original depots (i.e., Zhengzhou depot and Zhengzhoudong depot) to 10 and 30 respectively. Meanwhile, considering open potential depots with a maximum budget that is 30 tracks budget for each new potential depot. Key statistics and depot information are presented in Tables 4 and 5.

The meaning of the columns in Tables 4 and 5 are the same as those in Tables 2 and 3. Also, the lines in italics in both Tables 4 and 5 represent information from the manual schedule. Compared to the scenario 1, the computation times of scenario 2 increase a lot due to the limitation on the number of depot tracks in the two original depots, as it is more difficult to obtain feasible solutions from stage 1. Note that if we only consider the two original depots with the limited capacities, no feasible solution can be obtained. Therefore, for cases 2 and 9, there is no statics and information. From the statistics in Table 4, we can see that the trend of objective value, deadheading mileage and the number of rolling stock units used are similar to those in scenario 1. There are nearly 1.2% of operation cost savings for all these cases. The depot “LYML” is still the first one considered to build. Different from scenario 1, depot tracks are needed for the depot “LYML”. The “SQ” depot is no longer the most important location among all these depot locations. When considering opening 4 depots (cases 4 and 11) in the railway network, two data-sets give different options. The “XCD” depot becomes an alternative choice of the “SQ” depot in data-set 2, which also undertakes most of the pressure among others when increasing the value of the maximum number of depots. In addition, the “JZ” depot is used in data-set 1, while it is not necessary for data-set 2.

Similarly, the computer-based plans generated from the data in Scenario 2 were better than the manual plans. In summary, if the capacity needs to be transferred from the two old depots to new ones, the “LYLM” depot could be the best choice to start. With more capital budget or considering longer-term planning, the “SQ” and “XCD” depots can be selected to better serve the railway network.

**Table 4:** Key statics for different cases of scenario 2.

**Data**	Index	Time	${Depot_{num}}$	RS	${L_{avg}}$	${L_{min}}$	${L_{max}}$	${L_{tdh}}$	${L_{adh}}$	OP	Obj
1	*1*	*–*	*2*	*57*	*2435*	*183*	*4898*	*5291*	*92.8*	*13*	*124,471*
	2	–	2	–	–	–	–	–	–	–	–
	3	1810s	3	51	2670.0	630	4858	4371	85.7	13	109,311
	4	1315s	4	53	2594.2	419	5134	4153	78.4	11	107,268
	5	1764s	5	53	2584.9	464	5134	3660	69.1	11	106,780
	6	1811s	6	53	2581.5	409	5206	3493	65.9	11	106,618
	7	1503s	7	53	2580.9	510	5134	3457	65.2	11	106,587
2	*8*	*–*	*2*	*70*	*2575.7*	*571*	*5229*	*5240*	*74.8*	*18*	*160,753*
	9	–	2	–	–	–	–	–	–	–	–
	10	6412s	3	65	2882.8	726	5499	8772	135.0	18	147,902
	11	13,024s	4	65	2792.6	686	5316	6800	104.6	18	145,935
	12	7592s	5	67	2707.0	631	5348	6666	99.5	16	144,475
	13	8991s	6	67	2702.4	704	5209	6342	94.7	16	144,157
	14	7935s	7	67	2702.5	480	5462	6342	94.7	16	144,157

Manual schedule values are in [italics].

**Table 5:** Depot and its track occupy information of scenario 2.

Index	${Depot_{use}}$	ZZ	ZZD	SQ	LYLM	XCD	JZ	AYD
*1*	*2*	*10*	*47*	*–*	*–*	*–*	*–*	*–*
2	–	–	–	–	–	–	–	–
3	3	10	30	–	11	–	–	–
4	4	10	30	3	10	–	–	–
5	5	10	30	3	5	5	–	–
7	6	10	30	3	5	4	–	1
7	7	10	30	3	5	2	2	1
*8*	*2*	*7*	*63*	*–*	*–*	*–*	*–*	*–*
9	–	–	–	–	–	–	–	–
10	3	10	30	–	25	–	–	–
11	4	10	30	–	5	20	–	–
12	5	10	30	3	5	19	–	–
13	6	10	30	3	5	17	–	2
14	6	10	30	3	5	17	–	2

Manual schedule values are in [italics].

Through the data analysis of the above two different scenarios, it can be seen that the location of Luoyang Longmen in the railway network has priority to select as the new location of the rolling stock depot, followed by the location of Shangqiu. According to the information released by the China Railway, the Luoyang Longmen rolling stock depot opened for operation on April 18, 2021, which has less capacity for maintenance and storage compared to other depots. The Shangqiu depot was officially planned in July 2021, and construction started in March 2023.

#### Sensitive analysis

Two kinds of operating scenarios are analyzed in the above two sections, which can provide useful information to the railway manager. During the calculation process, the parameters related to the construction cost were set to a fixed value. Generally, the setting of cost parameters has an important impact on the computational results. For example, in this paper, the cost of selecting a new depot decides whether the depot is open or not and the cost of building one depot track limits the number of tracks in a depot. In this paper, when it comes to the competition of construction costs and daily operating costs, there is a large magnitude difference between them. Therefore, we consider converting construction costs into generalized operating costs. According to the relevant investment documents of China Railway, the cost of building a new depot (with depot tracks) is about 100 million RMB to 150 million RMB. In addition, the recycling period of railway infrastructure is generally set to 25 years and A normal depot usually contains 10 depot tracks (storage track and maintenance track). Thus, if we convert the cost to 1 day of one depot track that is around 1000–1500 RMB. As shown in Table 3 in “Scenario 1” section, some rolling stock schedules only need the deadheading possibilities of a depot. In this case, the corresponding depot needs to be opened, but there is no need to build depot tracks inside it. Since a connecting track between the depot and the main line is required, the opening cost of a depot is set the same as the construction cost of a depot track. Besides, in Eq. (1), the trade-off relationship between the construction cost and the combined operating cost is affected by weight G. To further explore the impact of construction cost on the results of the paper, different parameter settings are analyzed in this section, as shown in Table 6.

**Table 6:** Different parameter settings of sensitive analysis.

Index	*G*	${C_d^{trc}}$	${C_d^{dpot}}$
1	0.005	5000	1000
2	0.005	10,000	1000
3	0.005	50,000	1000
4	0.005	1000	5000
5	0.005	1000	10,000
6	0.005	1000	50,000
7	0.05	1000	1000
8	0.2	1000	1000
9	0.5	1000	1000

To better explore the impact of the above parameters on the conclusion of the paper, the data-1 of scenario 1 with 7 depots open is selected here. The key statics of the sensitive analysis is shown in Table 7 and the corresponding depot(track) information is shown in Table 8.

**Table 7:** Key statics for different settings of sensitive analysis.

Index	*G*	${C_d^{trc}}$	${C_d^{dpot}}$	RS	${L_{avg}}$	${L_{min}}$	${L_{max}}$	${L_{tdh}}$	${L_{adh}}$	OP	Obj
1	0.005	5000.0	1000.0	53	2571.8	469	5134	2969	56	11	106,079
2	0.005	10,000.0	1000.0	53	2571.8	513	5134	2969	56	11	106,129
3	0.005	50,000.0	1000.0	53	2571.8	469	5134	2969	56	11	106,529
4	0.005	1000.0	5000.0	53	2572.1	589	5134	2993	56.5	11	106,098
5	0.005	1000.0	10,000.0	53	2572.1	364	5134	2993	56.5	11	106,148
6	0.005	1000.0	50,000.0	53	2572.3	421	5134	2993	56.5	11	106,548
7	0.050	1000.0	1000.0	53	2572.3	364	5134	3001	56.6	11	106,241
8	0.200	1000.0	1000.0	53	2572.4	364	5134	3001	56.6	11	106,481
9	0.500	1000.0	1000.0	53	2586.5	469	5321	3749	70.7	11	107,789

**Table 8:** Key Statics for Different Settings of Sensitive Analysis.

Index	*G*	${C_d^{trc}}$	${C_d^{dpot}}$	ZZ	ZZD	SQ	LYLM	XCD	JZ	AYD
1	0.005	5000	1000	17	34	2	0	0	–	0
2	0.005	10,000	1000	17	34	2	0	0	–	0
3	0.005	50,000	1000	17	34	2	0	0	–	0
4	0.005	1000	5000	18	32	3	0	–	–	–
5	0.005	1000	10,000	18	32	3	0	–	–	–
6	0.005	1000	50,000	18	32	3	0	–	–	–
7	0.05	1000	1000	18	33	2	0	–	–	–
8	0.2	1000	1000	18	33	2	0	–	–	–
9	0.5	1000	1000	18	35	0	0	–	–	–

A comparison of rows 1, 2, and 3 in Table 8 with row 7 in Table 3 shows that the number of tracks constructed in the Shangqiu depot decreases when the cost of constructing tracks in the depot is increased; Also comparing the information in rows 1, 2, and 3 of Table 7 with the information in row 7 of Table 2 reveals an increase in deadheading mileages after increasing the cost of building depot track. Similarly, when the cost of opening a depot is increased, a comparison of rows 4, 5, and 6 of Table 8 with row 7 of Table 3 shows that two fewer depots are opened, i.e., no more XCD depot and no more AYD depot. In addition, based on the information in rows 7, 8, and 9 of Table 7 and Table 8, it can be seen that when the cost of opening a depot and building a depot track are increased at the same time, the number of depots opened decreases and the number of new tracks built decreases as well. The synthesis of the above analysis shows that the construction cost has a direct impact on the construction of depots (depot tracks). It also reflects that different proposed strategies can be obtained by flexibly setting the construction cost parameters according to the construction cost demand in the construction process.

## Conclusions

With the fast expanding of CHSR, the coordination between the capacity of settled facilities and transportation needs should improve. The building of new depots or rebuilding old depots become one of the economical and practical methods. This paper presents a two-stage approach for exploring the joint depot location, capacity and rolling stock scheduling optimization with maintenance requirements. Two MILP problems form the core of our approach. The MILP model in the first stage generates multiple candidate rolling stock schedules with multiple depot location and capacity options, and the MILP model in the second stage is responsible for checking the maintenance feasibility. A real-world instance of two different operation scenarios provided by the Zhengzhou HSR network has been tested. Compared to the current manual schedule, there are significant operation cost savings for both scenarios. For scenario 1, there is about 2.4% of operating cost savings to serve 1 day timetable compared to the cases using two original depots. For scenario 2, it is possible to move capacities to the new depots with nearly 1.2% of operation cost savings.

Several interesting directions can be extended for further research. First, multiple timetables can be tested for offering more useful information. Also, it would be interesting to see the performance of the proposed approach to more different timetables. Second, the computation times could be further reduced by improving the algorithm efficiency. Although this is not a real-time problem, a fast algorithm is more practical and easier accepted by the railway company. Finally, the work could be extended to consider the integrated optimization of train timetabling,crew scheduling and energy consumpition^24–28^ and rolling stock scheduling with possible more system-wide benefits.

Moreover, the construction work of a depot need to be carried out step by step in practice, all these test cases in scenario 1 and 2 can provide useful information on different planning periods for the Zhengzhou Railway.

## Data Availability

The datasets analyzed during the current study are not publicly available due to confidential company data by Zhengzhou Railway but are available from the corresponding author upon reasonable request.
